# Associations Between Cancer-Related Fatigue and Healthcare Use During Cancer Follow-Up Care: A Survey-Administrative Health Data Linkage Study

**DOI:** 10.3390/curroncol31110542

**Published:** 2024-11-19

**Authors:** Robin Urquhart, Cynthia Kendell, Lynn Lethbridge

**Affiliations:** 1 Department of Community Health and Epidemiology, Dalhousie University, Halifax, NS B3H 1V7, Canada; 2 Department of Surgery, Dalhousie University/Nova Scotia Health, Halifax, NS B3H 2Y9, Canada; lynn.lethbridge@dal.ca; 3 Department of Medicine, Dalhousie University/Nova Scotia Health, Halifax, NS B3H 2Y9, Canada; cynthia.kendell@nshealth.ca

**Keywords:** cancer-related fatigue, survivorship, follow-up care, survey, administrative health data

## Abstract

Little is known about the impacts of fatigue after cancer treatment, including whether cancer-related fatigue impacts people’s use of healthcare. This study sought to examine how cancer-related fatigue impacts healthcare use after completing cancer treatment. A population-based survey was administered in Nova Scotia, Canada, to examine survivors’ experiences and needs after completing cancer treatment. Respondents included survivors of breast, melanoma, colorectal, prostate, hematologic, and young adult cancers who were 1–3 years post-treatment. Survey responses were linked to cancer registry, physicians’ claims, hospitalization, and ambulatory care data. Data were analyzed descriptively and using regression models. The final study cohort included 823 respondents. Younger respondents reported higher levels of cancer-related fatigue compared to older respondents. More females than males reported cancer-related fatigue. Upon adjusted analyses, those with cancer-related fatigue had lower odds of being discharged to primary care for their cancer-related follow-up (odds ratio = 0.71, *p* = 0.029). Moreover, those with cancer-related fatigue had 19% higher primary care use (incidence rate ratio = 1.19, *p* < 0.0001) and 37% higher oncology use (incidence rate ratio = 1.37, *p* < 0.016) during the follow-up period compared to those without cancer-related fatigue. Providers (oncology and primary care) may require additional support to identify clinically relevant fatigue and refer patients to appropriate resources and services.

## 1. Introduction

Worldwide, the prevalence of cancer survivors is increasing due largely to advances in early detection and cancer therapies [1,2]. In Canada alone, more than 1.5 million people are living with or beyond cancer [3]. As these individuals move beyond their cancer treatment, many will experience ongoing, persistent needs that negatively impact their health-related quality of life as well as their reintegration into work and social roles and activities [4,5]. Cancer-related fatigue is one of the most commonly reported ongoing effects of a cancer diagnosis and its treatment [4,6,7,8]. A large Canadian study of cancer survivors’ experiences and needs found that cancer-related fatigue was the most prevalent need of survivors who had completed cancer treatment 1–3 years prior, with 67% reporting it as an ongoing concern [4]. Defined as “a distressing, persistent, subjective sense of physical, emotional, and/or cognitive tiredness or exhaustion related to cancer or cancer treatment that is not proportional to recent activity and interferes with usual functioning” [9], cancer-related fatigue can endure for years after a person has completed cancer treatment, with 25–40% of people reporting persistent fatigue up to 10 years after treatment completion [7,10,11,12,13,14,15]. For many people, cancer-related fatigue interferes with reintegration processes and negatively impacts health-related quality of life [16,17,18,19].

Despite its high prevalence and the existence of effective, evidence-based management options [20,21,22], cancer-related fatigue is not well managed in the growing population of people living with and beyond cancer. This may be because healthcare providers do not inquire about or assess for fatigue [23] or may not appreciate its impact on their patient’s recovery and well-being [24,25,26]. Further, patients and survivors may be hesitant to report their fatigue during routine visits because they feel nothing can be done to manage it [27]. Although the prevalence of cancer-related fatigue is well documented, much less is known about the impacts of fatigue after cancer treatment, including whether cancer-related fatigue impacts people’s use of the healthcare system. Such knowledge can help delineate the impact of fatigue and point to potential places for intervention. In this study, we sought to examine cancer-related fatigue and healthcare use during post-treatment and follow-up care.

## 2. Materials and Methods

### 2.1. Study Design and Population

This study involved a linkage of cross-sectional survey data to population-based administrative health data. The study cohort consisted of survivors of breast, colorectal, prostate, melanoma, hematological, and adolescent and young adult (AYA) cancers in Nova Scotia, Canada, who were 1–3 years post-treatment, responded to the population-based survey, remained cancer-free during the four-year follow-up period, and did not die during the study period. Respondents also had to have had a visit with a cancer specialist at any time point post-diagnosis. Ethical approval to conduct this study was granted by the Nova Scotia Health Research Ethics Board.

### 2.2. Data Sources

The source of survey data was the “Cancer Transitions Survey”, a population-based survey administered in all 10 Canadian provinces in 2016. The survey included 83 items that focused on survivors’ experiences and needs after completing cancer treatment. It was informed by two validated surveys [28,29] and then expanded after consultation with subject matter experts, provincial leads, patients/survivors, and an expert panel. Further details about survey development and testing are reported elsewhere [4]. The results of this national study have been published [4,30,31].

In Nova Scotia, Canada, the survey was administered by the Nova Scotia Cancer Registry (NSCR). The survey was sent to all Nova Scotia cancer survivors who met certain eligibility criteria. Table 1 details the eligibility criteria for those aged 30+ at diagnosis. The stage at diagnosis for solid tumor cancers was based on the Collaborative Stage system, a unified data collection system combining clinical and pathologic data to derive a tumor (T), node (N), and metastasis (M) stage. For those aged 18–29 at diagnosis, individuals were diagnosed between 2 May 2012 and 2 May 2014 with a primary diagnosis of any invasive cancer (behavior code = 3). The following exclusion criteria were applied: (1) stage IV at diagnosis (except for testicular cancer), (2) non-melanoma skin cancer, (3) Kaposi’s sarcoma, and (4) cases recorded as having died at the time of cohort extraction.

The NSCR sent potential participants a personalized invitation letter, an information sheet, and a hard copy of the survey package via mail. All individuals invited to participate were assigned a unique barcode number and PIN. Surveys could be returned via mail or completed online without requiring participants to provide identifying information using this approach. The survey was open for a 6-week data collection period. Reminder letters were sent at approximately 28 days post-survey launch. No identifying information was collected from participants.

All participants provided consent to link their survey data to administrative health databases for use in subsequent research. A study “key” linking each of the barcodes and PINs to identifiable individuals was created and held by the NSCR. This file permitted the NSCR to append health card numbers to the survey dataset to facilitate subsequent linkages.

For this study, the survey dataset was linked to the following administrative health datasets: NSCR (to obtain data on cancer type, diagnosis date, stage at diagnosis, date of death, and cancer history); Oncology Patient Information System (to obtain data on treatments received and variables for determining disease recurrence); MSI Insured Patient Registry (to determine enrolment in the provincial insurance health program); MSI Physician Billings (to obtain claims data, including provider specialty, dates of visits, diagnoses, procedure codes); and the Canadian Institute of Health Information (CIHI) Discharge Abstract Database (to obtain hospitalization dates, diagnoses, and procedures indicating cancer recurrence) and National Ambulatory Care Reporting System (to obtain visit data for ambulatory care clinics and emergency department use). We obtained data from all datasets until 31 December 2019 to ensure four full years of follow-up data for each cancer survivor.

### 2.3. Analyses

The follow-up period was defined as beginning one year after the diagnosis date in the NSCR and continuing for four years (the end of the study period). The outcomes were (1) discharge from specialist to primary care for cancer-related follow-up (binary data; yes/no) and (2) the number of follow-up visits to oncology specialists (medical, radiation, and surgical oncology) and the number of follow-up visits to primary care (count data; “cancer-specific”, “non-cancer”, and “all” visits). Discharge was defined as zero visits to a specialist in the follow-up period. Cancer-specific visits were defined as any visit containing a diagnosis code corresponding to “neoplasm” (i.e., ICD-9 codes 140 to 239 inclusive). All outcomes were derived using administrative health data. The covariates were age at diagnosis, sex (male, female), chronic conditions (0, 1+), disease site (breast, colorectal, prostate, melanoma, hematological, and other), stage at diagnosis (I, II, III), place of residence (rural, urban), place of birth (Canada, other), highest education attained (less than a high school diploma, high school, some post-secondary, certificate/diploma, degree), speaks English or French most often (yes, no), marital status (married/partnered or not), and cancer-related fatigue (yes/no, where yes = moderate/big cancer-related fatigue and no = low/no cancer-related fatigue). All covariates, except disease site, age at diagnosis, stage at diagnosis, and place of residence, were obtained from the survey. Rural and urban status were determined based on the postal code at diagnosis, where a second digit of 0 indicates rural residence. Disease site, stage at diagnosis, age at diagnosis, and place of residence (using postal code at diagnosis) were obtained from the NSCR.

Descriptive statistics were computed to describe the study cohort, stratified by those who reported cancer-related fatigue and those who did not. Multivariable logistic regression models were run to test whether cancer-related fatigue was associated with discharge to primary care. To test whether cancer-related fatigue was associated with the number of physician visits (oncologist and primary care) after cancer treatment, multivariable Poisson regression was used, with negative binomial regression for outcomes that had an overdispersion of zeros. Odds ratios (OR) and incidence rate ratios (IRR) were reported, and a confidence level of 95% was used to determine statistical significance. SAS version 9.4 was used to complete all analyses.

## 3. Results

The final study cohort was cancer survivors who responded to the survey, had their data linked, completed the cancer-related fatigue survey items, were cancer-free during the four-year follow-up period, and saw an oncology specialist at least once post-diagnosis (*n* = 823). 399 of 823 respondents (48.5%) reported experiencing cancer-related fatigue at the time of survey completion. Table 2 presents the socio-demographic and disease characteristics of the cohort, stratified by those who reported cancer-related fatigue and those who did not. In brief, the mean age at diagnosis of those who reported cancer-related fatigue was 61.3 years, while the mean age of those who did not report cancer-related fatigue was 63.5 years. Of those who reported cancer-related fatigue, 64.2% were female, 15.3% had at least one other chronic condition, and 46.6% had breast cancer. Since all breast cancer patients were female and 64.2% were female, this indicates that 72.7% of females who had fatigue had breast cancer.

Cancer-related fatigue was associated with discharge to primary care (Table 3), whereby those who experienced cancer-related fatigue had reduced odds of discharge (OR = 0.71; 95%CI = 0.52–0.97). Table 4 and Table 5 present the incidence rate ratios for primary care and specialist visits, respectively. Upon adjusted analyses, cancer-related fatigue was associated with higher primary care and specialist use during follow-up care. Compared to those without cancer-related fatigue, those with cancer-related fatigue had a 19% higher rate of all primary care visits (IRR = 1.19, 95%CI = 1.16–1.22) and a 19% higher rate of non-cancer-related primary care visits (IRR = 1.19, 95%CI = 1.16–1.23) during follow-up care. Similarly, those with cancer-related fatigue had a 37% higher rate of cancer specialist visits during follow-up care compared to those without cancer-related fatigue (IRR = 1.37, 95%CI = 1.06–1.77).

## 4. Discussion

This study sought to understand whether and how cancer-related fatigue is associated with healthcare use during follow-up care. Nearly 50% of respondents reported cancer-related fatigue at the time of survey completion (1–3 years post-cancer treatment). Those who reported cancer-related fatigue were less likely to be discharged to primary care for follow-up care and had more visits to primary care and specialist care during the follow-up period when compared to people who reported no cancer-related fatigue. These findings suggest there are many potential points of intervention and that both oncology and primary care providers may benefit from additional education and resources around cancer-related fatigue so they can better support their patients’ recovery from cancer.

The prevalence of cancer-related fatigue in cancer survivors is well documented, although few researchers have studied how fatigue may impact people’s ongoing use of the health system. The findings from this study, which demonstrated that individuals with cancer-related fatigue had higher use of primary and specialist care during the follow-up period, align with the research that has been done [32,33]. Jones and colleagues [32] found that early-stage breast and colorectal cancer survivors who were within five years of treatment completion and who experienced clinically significant cancer-related fatigue reported higher use of physicians and other health professionals compared to those who did not experience clinically significant fatigue. Moreover, a study of primary care use in the Netherlands found that breast, colorectal, and prostate cancer survivors, 2–5 years after diagnosis, visited their primary care providers more often for fatigue than age- and sex-matched non-cancer controls [34]. We found no studies examining whether ongoing cancer-related fatigue is related to discharge back to primary or community care after cancer treatment. In this study, those reporting cancer-related fatigue were less likely to be discharged to primary care (and therefore more likely to remain with their specialist team). Given the negative impact on recovery and health-related quality of life [16,17,18,19], survivors with cancer-related fatigue may not feel “well enough” to return to primary care and worry that discharge would result in lower access to the support and services they need to recover. Indeed, cancer-related fatigue often co-occurs with other ongoing symptoms, such as insomnia, pain, appetite loss, and depression [35,36]. Earlier identification and management of cancer-related fatigue by the cancer care team may reduce some of these other symptoms [35] and enable a smoother discharge from specialists to primary care after cancer treatment.

Cancer-related fatigue is a prevalent and particularly problematic concern for cancer survivors. Cancer-related fatigue has been reported as the most disruptive ongoing effect during the cancer survivorship period [37]. Therefore, early identification and management of cancer-related fatigue is imperative to helping people optimally recover and live well beyond their cancer diagnosis and treatment. Early identification is particularly germane, given there are effective options for managing cancer-related fatigue [20,21,22]. Unfortunately, cancer-related fatigue often remains unrecognized and unasked about in clinical practice [23,24,25]. Yet, given the increased contact that those with cancer-related fatigue have with their physicians, many opportunities exist to assess and intervene. Physicians and the broader care team may require additional education or support to assess cancer-related fatigue and recognize its impact on their patients’ recovery after cancer [25].

This study has several key strengths. One is the linkage of survey data to administrative health data, which provides a more comprehensive and patient-centered opportunity to understand patterns of healthcare use during the follow-up care period. Related to the linkage of these data sources was our ability to objectively capture data on cancer type, diagnosis date and stage, and disease status (though linkage with the NSCR), which may not have been accurately captured in the self-reported survey dataset. Nevertheless, this study also has several limitations that must be highlighted when considering the findings. First, the survey was not validated. However, it was based on two previously validated surveys [28,29] and was pilot-tested with >100 pilot participants. Second, the assessment of cancer-related fatigue was not based on a validated scale but rather a question asking people to rate the extent to which cancer-related fatigue was a concern. While the use of a validated measure would have been ideal, the survey asked respondents about 20 specific needs post-treatment (e.g., insomnia, cognitive function, sexual dysfunction, fear of recurrence, depression, anxiety, among others); the use of validated measures to assess each of these needs would have greatly increased the length of the survey for respondents. Despite this, the prevalence of cancer-related fatigue in this study was within the range reported by others who have used validated scales. Finally, administrative data are only able to capture physician visits and not visits to other healthcare providers (e.g., nurses, psychologists, and allied health professionals) who may also play important roles in cancer survivors’ care after treatment.

## 5. Conclusions

Cancer-related fatigue is a highly prevalent ongoing concern for cancer survivors during the follow-up care period. This study demonstrates that those who report cancer-related fatigue visit their primary care and oncology providers more in the first four years of follow-up care than those who do not experience cancer-related fatigue. It also demonstrates that those with ongoing fatigue have lower odds of being discharged to primary care after cancer treatment. The higher level of physician use suggests there is ample opportunity to assess for cancer-related fatigue and intervene with management options. This includes counseling around or referral to exercise programs, cognitive behavioral therapy, and mindfulness-based programs [22]. Providers may require additional support to more effectively identify cancer-related fatigue in the growing cancer survivor population.

## Figures and Tables

**Table 1:** Respondents 30+ years of age at diagnosis: inclusion and exclusion criteria by disease site.

Disease Site	Timeframe	Inclusions	Exclusions
Breast	2 May 2012 to 2 May 2014	ICD-O-3 topography code C50.0 to C50.9 (inclusive) Behaviour code = 3 Female breast cancer cases only	Stage IV at diagnosis Lymphoma M95 to M98 (inclusive) Sarcomas Cases recorded as having died (at the time of extraction)
Colorectal	2 May 2012 to 2 May 2014	ICD-O-3 topography codes: C18.0, C18.2 to C18.9, C19.9, C20.9 and C26.0 Behaviour code = 3	Stage IV at diagnosis Lymphoma codes M-95 to M-98 (inclusive) Sarcomas Cases recorded as having died (at the time of extraction)
Prostate	2 May 2012 to 2 May 2014	ICD-O-3 topography code C61.9 Behaviour code 3	Stage IV at diagnosis Cases recorded as having died (at the time of extraction) ICD-O-3 histology codes: 9050–9055, 9140 and 9590–9992
Melanoma	2 November 2012 to 2 November 2014	ICD-O-3 topography code C44 ICD-O-3 histology codes 8720 to 8790 (inclusive) Behaviour code = 3	Stage IV at diagnosis Cases recorded as having died (at the time of extraction)
Hodgkin Lymphoma	2 August 2012 to 2 August 2014	≥30 years of age at diagnosis ICD-O-3 histology codes: 9650–9655, 9659, 9661–9665, 9667	Hodgkin Lymphoma and Diffuse Large B-Cell Lymphoma: Stage IV (Cotswold Staging System), Stage IV (Ann Arbor Staging System), or collaborative stage IV at diagnosis Cases recorded as having died (at the time of extraction)
Diffuse B-cell lymphoma	2 August 2012 to 2 August 2014	ICD-O-3 histology codes: 9680	
Acute myelogenous leukemia	2 August 2012 to 2 August 2014	≥30 years of age at diagnosis ICD-O-3 histology codes: 9840, 9861, 9865–9867, 9869, 9871–9874, 9895–9897, 9898, 9910–9911, 9920	
Acute lymphocytic leukemia	2 May 2010 to 2 May 2012	≥30 years of age at diagnosis ICD-O-3 histology codes: 9826, 9835–9836 For the histology codes 9811–9818 and 9837, topography codes C420, C421 and C424 were applied	

**Table 2:** Cohort characteristics (*n* = 823).

	Cancer-Related Fatigue (*n* = 399)	No/Low Cancer-Related Fatigue (*n* = 424)
Age at diagnosis (mean)	61.3 years	63.5 years
Sex		
Male	35.8%	55.9%
Female	64.2%	44.1%
Cancer type		
Hematologic	5.8%	1.9%
Breast ^1^	46.6%	28.5%
Colorectal	19.8%	20.1%
Melanoma	2.5%	14.6%
Prostate ^2^	23.1%	33.3%
Other ^3^	2.3%	1.7%
Stage at diagnosis		
Stage I	35.1%	46.7%
Stage II	39.6%	37.5%
Stage III	25.3%	15.8%
Rural residence	35.6%	34.9%
Any chronic condition	15.3%	9.7%
Education attained		
Less than a high school diploma	19.1%	18.2%
High school diploma	20.6%	18.6%
Some post-secondary education	19.8%	19.1%
Post-secondary certificate	17.0%	19.1%
Post-secondary degree	8.5%	10.6%
Speaks English or French	99.8%	99.5%
Born in Canada	93.3%	91.8%

^1^ All breast cancer patients are female. ^2^ All prostate cancer patients are male. ^3^ Other category = patients aged 18–29 at diagnosis with a primary diagnosis of any invasive cancer, excluding (1) stage IV at diagnosis (except for testicular cancer); (2) non-melanoma skin cancer; and (3) Kaposi’s sarcoma.

**Table 3:** Odds Ratios for discharge to primary care. Significant (*p* < 0.05) results are bolded.

Variable	Discharge to Primary Care Logistic Regression		
	Odds Ratio	Lower CI	Upper CI
Cancer-related fatigue	**0.71**	**0.52**	**0.97**
Male sex	0.94	0.55	1.61
Married or partnered	1.18	0.81	1.70
Any chronic condition	0.80	0.51	1.26
Age at diagnosis (reference: 55–64 years)			
18–54 years	1.18	0.57	2.44
65–74 years	0.63	0.38	1.04
75+ years	**0.27**	**0.11**	**0.67**
Disease site (reference: other)			
Hematological	0.57	0.11	2.94
Breast	1.66	0.42	6.6
Colorectal	1.61	0.41	6.34
Melanoma	**6.92**	**1.63**	**29.34**
Prostate	2.11	0.54	8.30
Stage at Diagnosis (reference: stage I)			
Stage II	**0.62**	**0.43**	**0.88**
Stage III	**0.50**	**0.32**	**0.77**
Rural residence	1.28	0.93	1.76
Born in Canada	1.66	0.89	3.09
Education attained (reference: less than high school)			
High school	1.27	0.82	1.98
Some post-secondary	1.25	0.82	1.91
Post-secondary certificate/diploma	**1.67**	**1.09**	**2.56**
Post-secondary degree	1.64	0.94	2.87
Speaks English or French	0.73	0.06	8.99

**Table 4:** Incidence Rate Ratios (adjusted analyses) for primary care visits during follow-up care. Significant (*p* < 0.05) results are bolded.

Variable	Primary Care Visits (All) Poisson			Primary Care Visits (Cancer-Related) Negative Binomial			Primary Care Visits (Non-Cancer-Related) Poisson		
	IRR	Lower CI	Upper CI	IRR	Lower CI	Upper CI	IRR	Lower CI	Upper CI
Cancer-related fatigue	**1.19**	**1.16**	**1.22**	1.18	0.98	1.42	**1.19**	**1.16**	**1.23**
Male sex	**0.92**	**0.88**	**0.97**	0.91	0.67	1.25	**0.93**	**0.89**	**0.98**
Married or partnered	0.98	0.95	1.01	1.16	0.93	1.44	**0.96**	**0.93**	**0.99**
Any chronic condition	**1.24**	**1.20**	**1.29**	1.18	0.90	1.54	**1.24**	**1.20**	**1.29**
Age at diagnosis (reference: 55–64 years)									
18–54 years	**0.82**	**0.77**	**0.88**	0.74	0.48	1.16	**0.81**	**0.76**	**0.88**
65–74 years	**1.17**	**1.12**	**1.22**	1.21	0.91	1.61	**1.16**	**1.11**	**1.22**
75+ years	**1.48**	**1.37**	**1.59**	**1.71**	**1.04**	**2.79**	**1.47**	**1.36**	**1.58**
Disease site (ref: other)									
Hematological	**0.76**	**0.68**	**0.84**	**0.46**	**0.21**	**0.99**	**0.84**	**0.75**	**0.95**
Breast	**0.75**	**0.69**	**0.82**	0.75	0.39	1.43	**0.78**	**0.71**	**0.87**
Colorectal	**0.81**	**0.74**	**0.88**	0.80	0.42	1.54	**0.84**	**0.76**	**0.93**
Melanoma	**0.68**	**0.61**	**0.75**	**0.20**	**0.10**	**0.41**	**0.79**	**0.71**	**0.88**
Prostate	**0.79**	**0.72**	**0.86**	0.68	0.35	1.32	**0.83**	**0.75**	**0.92**
Stage at Diagnosis (ref: stage I)									
Stage II	0.99	0.96	1.02	1.05	0.85	1.30	0.98	0.95	1.01
Stage III	1.02	0.98	1.06	1.08	0.83	1.39	1.00	0.96	1.04
Rural residence	**0.91**	**0.88**	**0.93**	0.94	0.78	1.14	**0.90**	**0.87**	**0.92**
Born in Canada	**0.95**	**0.91**	**1.00**	0.94	0.65	1.35	**0.94**	**0.89**	**0.99**
Education attained: (Ref: Less than high school)									
High school	**1.19**	**1.15**	**1.23**	1.09	0.84	1.42	**1.20**	**1.15**	**1.25**
Some post-secondary	**1.07**	**1.03**	**1.11**	0.82	0.64	1.05	**1.12**	**1.08**	**1.16**
Post-secondary certificate	1.03	0.99	1.07	0.95	0.73	1.22	1.04	1.00	1.08
Post-secondary degree	0.99	0.94	1.04	**0.64**	**0.45**	**0.91**	1.04	0.99	1.09
Speaks English or French	**0.72**	**0.60**	**0.86**	1.30	0.30	5.68	**0.67**	**0.55**	**0.81**

**Table 5:** Incidence Rate Ratios for oncology visits during follow-up care. Significant (*p* < 0.05) results are bolded.

Variable	Oncology Visits Negative Binomial		
	IRR	Lower CI	Upper CI
Cancer-related fatigue	**1.37**	**1.06**	**1.77**
Male sex	1.25	0.79	1.97
Married or partnered	0.92	0.69	1.24
Any chronic condition	1.16	0.80	1.68
Age at diagnosis (reference: 55–64 years)			
18–54 years	0.68	0.38	1.23
65–74 years	1.26	0.84	1.89
75+ years	**2.68**	**1.34**	**5.34**
Disease site (reference: other)			
Hematological	1.79	0.64	5.03
Breast	0.93	0.36	2.38
Colorectal	**0.28**	**0.11**	**0.71**
Melanoma	**0.26**	**0.10**	**0.71**
Prostate	**0.43**	**0.17**	**1.07**
Stage at Diagnosis (reference: stage I)			
Stage II	**1.48**	**1.09**	**2.01**
Stage III	**2.47**	**1.72**	**3.56**
Rural residence	**0.68**	**0.52**	**0.89**
Born in Canada	**0.59**	**0.36**	**0.97**
Education attained (reference: less than high school)			
High school	1.11	0.77	1.60
Some post-secondary	0.96	0.68	1.35
Post-secondary certificate/diploma	0.84	0.59	1.21
Post-secondary degree	**0.46**	**0.29**	**0.75**
Speaks English or French	4.34	0.50	37.73

## Data Availability

The datasets presented in this article are not readily available because of privacy and ethics restrictions. Requests to access the datasets should be directed to Robin Urquhart.
